# Optical Coherence Tomography Angiography of Macula and Optic Nerve in Autism Spectrum Disorder: A Pilot Study

**DOI:** 10.3390/jcm9103123

**Published:** 2020-09-27

**Authors:** Jose Javier Garcia-Medina, Elena Rubio-Velazquez, Maria Dolores Lopez-Bernal, Dolores Parraga-Muñoz, Alfonso Perez-Martinez, Maria Dolores Pinazo-Duran, Monica del-Rio-Vellosillo

**Affiliations:** 1 Department of Ophthalmology, General University Hospital Morales Meseguer, 30008 Murcia, Spain; elena.rubio@carm.es (E.R.-V.); dolores.lopez11@carm.es (M.D.L.-B.); lolika_13pm@hotmail.es (D.P.-M.); 2 Department of Ophthalmology and Optometry, University of Murcia, 30120 Murcia, Spain; 3 Ophthalmic Research Unit Santiago Grisolia, 46017 Valencia, Spain; dolores.pinazo@uv.es; 4 Red Temática de Investigación Cooperativa en Patología Ocular (OFTARED), Instituto de Salud Carlos III, 28029 Madrid, Spain; 5 Department of Clinical Analysis, General University Hospital Morales Meseguer, 30008 Murcia, Spain; alfonso.perez3@carm.es; 6 Department of Ophthalmology, University of Valencia, 46010 Valencia, Spain; 7 Department of Anesthesiology, University Hospital Virgen de la Arrixaca, 30120 Murcia, Spain; monicadelriov@hotmail.com

**Keywords:** autism spectrum disorder, neurotypical, optical coherence tomography angiography, macula, optic nerve, thickness, perfusion, flux, vessel density, autoregulation

## Abstract

The aim of this study was to compare retinal thicknesses and vascular parameters between autism spectrum disorder (ASD) and neurotypical (NT) individuals. Recruited ASD subjects and age- and sex-matched NT controls underwent 2 optical coherence tomography scans (OCT) (macular cube and optic nerve cube) and 2 OCT angiography (OCTA) scans (macular and optic nerve head (ONH) OCTA) with the device Cirrus 5000 (Zeiss). Concerning OCT, we considered full retina thickness in 9 macular sectors of the Early Treatment Diabetic Retinopathy Study (ETDRS) pattern and peripapillary retinal nerve fiber layer (pRNFL) thickness in 4 quadrants and 12 clock-hour sectors. Vessel density and capillary perfusion density in 9 sectors were measured using 6 × 6 mm macular OCTA. Foveal avascular zone (FAZ) parameters were also considered. ONH 4.5 × 4.5 mm OCTA estimated perfusion density and flux index in 4 peripapillary quadrants. Comparisons between groups of all these parameters were performed. ASD subjects showed higher ONH perfusion density and lower ONH flux index at the peripapillary inferior quadrant when compared with NT individuals (*p* < 0.05). Plus, a trend towards higher macular thicknesses, higher pRNFL thickness at inferior clock-hour sectors and higher macular vessel density and perfusion was observed in ASD. No differences were found in FAZ parameters. In conclusion, retinas of ASD subjects may present some structural and vascular differences when compared with retinas of NT individuals.

## 1. Introduction

Autism spectrum disorder (ASD) is defined as a series of developmental disturbances in non-verbal and verbal communication, social skills and repetitive behavior [1]. Its prevalence is above 1% in all of the latest studies in different countries [2].

The etiology of this disorder remains elusive. Several causes and effects have been proposed [3]. Vascularization and blood flow in a number of regions of interest (ROI) have been studied in ASD. In general terms, hypoperfusion have been found in temporal [4,5,6,7,8,9,10,11], parietal [5,10,11] frontal [6,7,9,10,11,12,13] cortices and basal ganglia [8,11,13]. In contrast, hyperperfusion in the frontal and temporal cortices has also been described in ASD subjects in other investigations [14,15].

Developmentally and histologically, the retina constitutes an extension of the central nervous system (CNS) [16]. Our group has previously studied retinal thickness by means of optical coherence tomography (OCT) in ASD concluding that ASD presented some thickened locations when compared to neurotypical (NT) individuals [17]. However, to the best of our knowledge, the retinal vascularization and perfusion have not been previously studied in the context of ASD.

In the CNS, the chance of direct observation of microvascularization is only available for the retina vessels, which derive from the internal carotid artery through the ophthalmic artery [18].

A new technology called OCT angiography (OCTA) allows us to quantify vascular parameters in vivo in human retinas non-invasively and rapidly [19]. This is useful not only in ophthalmology but also in neuro-ophthalmology and neurology [20]. Because the retina is a part of the CNS, and considering the hypo and hyperperfusion found in other ROI, we hypothesized that retinal vascular parameters as measured by OCTA could be different in ASD when compared to NT individuals.

## 2. Materials and Methods

This prospective, cross-sectional study included participants between March and July 2019. Young ASD individuals were recruited from two specialized formation centers: Integral Formation Center ‘‘Gabriel Pérez Carcel’’ and ASTRADE, Murcia, Spain. NT controls were age- and sex-matched with ASD subjects and were selected among patients having habitual ophthalmic visits at the department of Ophthalmology, University Hospital Morales Meseguer, Murcia, Spain. All patients signed informed consent or, alternatively, parents/guardians’ consent was obtained. This study was approved by the Ethics Committee of the University Hospital Morales Meseguer, Murcia, Spain (Protocol Code: EST 10/19, 1st March 2019) and was in accordance with the declaration of Helsinki.

Inclusion criteria for ASD individuals were: (a) diagnosis of ASD following the 5th edition of the Diagnostic and Statistical Manual of Mental Disorders (DSM-5) criteria [21] assessed by at least two different professionals; (b) under 25 years of age; (c) of Caucasian race; (d) having less than 6 spherical diopters less than 6 and cylinder diopters less than 3 in absolute values; (e) best-corrected visual acuity (BCVA) of 0.5 in decimal scale or better; (f) no signs or records of eye disorders; (g) no other disease that may affect OCT or OCTA measurements; (h) cooperation to obtain high-quality and reliable OCT and OCTA examinations; (i) signal strength of OCT and OCTA of 6 or better (out of 10). The same experienced ophthalmologist (J.J.G.-M.) checked all the scans. If segmentation errors, decentrations or other artefacts were detected, examinations were considered unreliable.

Inclusion criteria for the NT participants were also selected using the above, except for criterium (a). Additionally, NT participants could not be relatives of ASD patients.

All ophthalmological examinations were carried out at the Department of Ophthalmology, University Hospital Morales Meseguer, Murcia, Spain and consisted of: autorefractometry, best-corrected visual acuity (BCVA), neumotonometry, biomicroscopy, fundus exploration, OCT and OCTA.

OCT and OCTA examinations were performed using Cirrus 5000 device with Angioplex (software version 11.0, Carl Zeiss Meditec, Dublin, CA, USA). Each participant had four scans in the left eye: Macular cube 512 × 128 OCT, optic nerve cube 200 × 200 OCT, macular OCTA 6 × 6 mm and optic nerve OCTA 4.5 × 4.5 mm.

**Figure 1:**
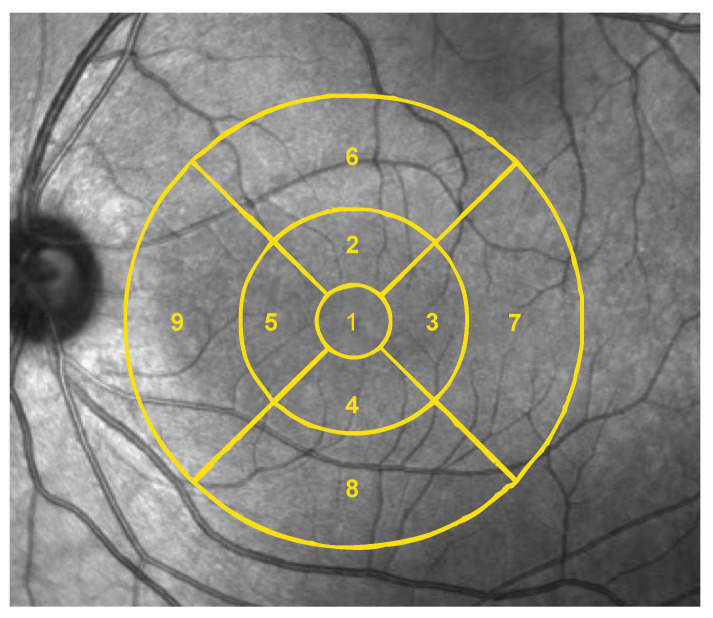
Early Treatment Diabetic Retinopathy Study (ETDRS) grid overlying the macula. Nine subfields are represented. 1 = center, 2 = inner superior, 3 = inner right, 4 = inner inferior, 5 = inner left, 6 = outer superior, 7 = outer right, 8 = outer inferior, 9 = outer left.

In macular cube OCT scans, the thickness of the complete retina (from internal limiting membrane to retinal pigment epithelium) was estimated at the nine subfields of the Early Treatment Diabetic Retinopathy Study (ETDRS) (Figure 1).

**Figure 2:**
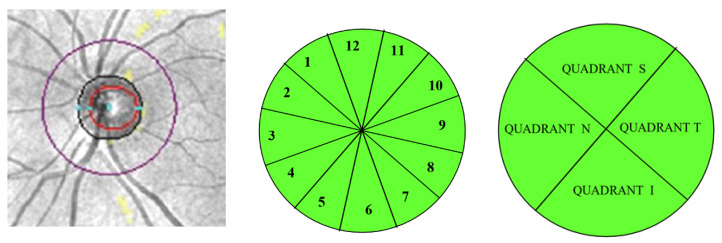
Peripapillary retinal nerve fiber layer was measured at the indicated purple circle (**left**). Clock-hour sectors (**center**) and quadrants (**right**) are represented. S = superior, N = nasal, T = temporal, I = inferior.

In optic nerve cube OCT scans, the thickness of the peripapillary retinal nerve fiber layer was measured in 12 clock-hour sectors and 4 quadrants (Figure 2).

The superficial capillary plexus, which extends from the internal limiting membrane to the inner plexiform layer, was measured by OCTA in macular scans.

**Figure 3:**
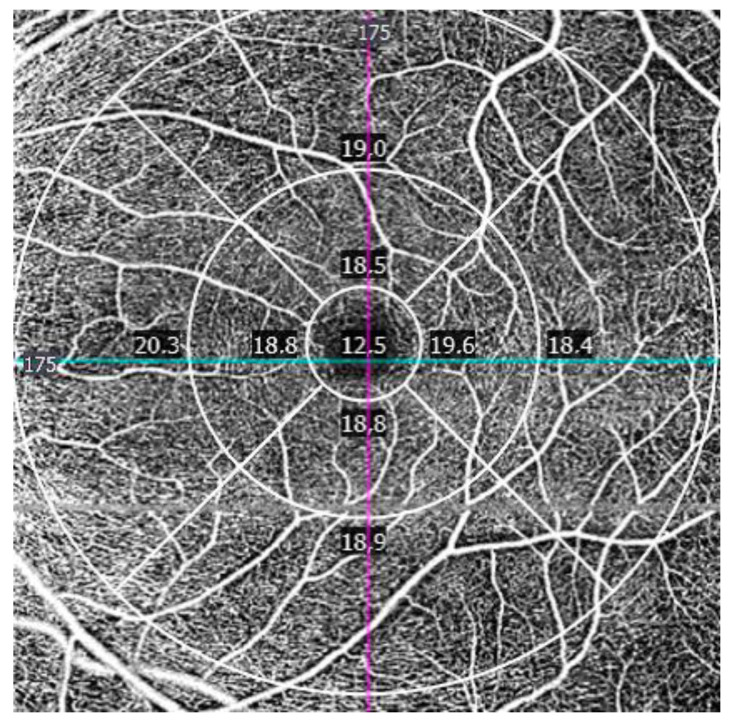
Vessel density map at the macula. Units are indicated in mm/mm^2^.

In macular OCTA, two vascular indices were automatically obtained using ETDRS grid (Figure 1): Vessel density (VD) and perfusion density (PD). VD is characterized as the absolute length of the perfused vasculature per area in a considered region of estimation. Its units are mm/mm^2^ ranging from 0 (no vessels) to an unbounded maximum (Figure 3). VD can be thought of as unwinding all the vasculature in a considered area, estimating its length with a ruler, then dividing the result by the involved area [22].

**Figure 4:**
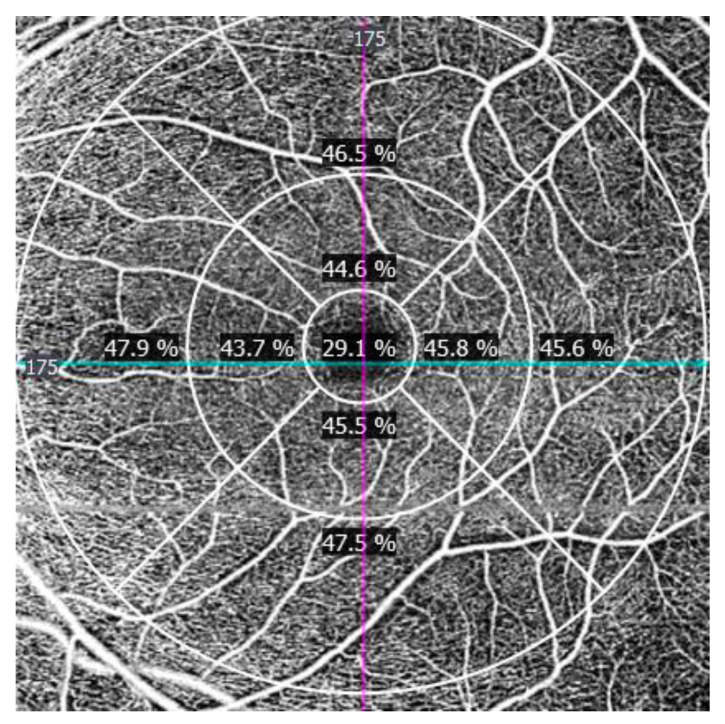
Perfusion density map at the macula. Units are unitless (%).

PD is characterized as the complete zone of perfused vasculature per unit of the considered area. This measurement is determined by summarizing the quantity of pixels which contain perfused vasculature and dividing this by the entirety of all pixels (perfused and non-perfused pixels) in the area. The outcome is a unitless number extending from 0% (no perfusion) to 100% (completely perfused) (Figure 4).

The primary distinction between VD and PD is that in VD all vessels are considered similarly. In PD, bigger vessels impact the estimation more than smaller vessels. Foveal avascular zone (FAZ) area, perimeter and circularity were also considered [22].

**Figure 5:**
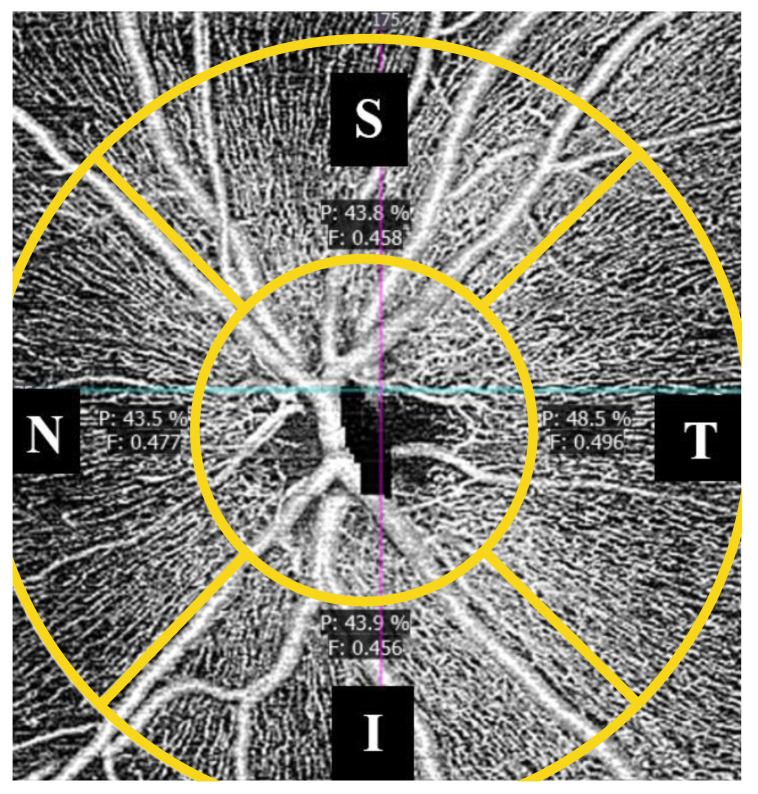
Peripapillary microvascularization map. Perfusion density (P) is indicated in percentage (%). Flux index (F) is unitless. S = superior, I = inferior, N = nasal, T = temporal.

In peripapillary OCTA, two vascular indices were also automatically obtained considering four quadrants (Figure 5) and measuring from internal limiting membrane to the retinal nerve fiber layer: peripapillary perfusion density (pPD) and flux index (FI). The pPD is defined, similarly to PD in the macula, as the total area of perfused vasculature per unit area in a region of interest, expressed in percentage. FI is defined as the total area of perfused vasculature per unit area in a considered region, weighted by the brightness (intensity) of the flow signal. FI quantifies the number of blood cells passing through a retinal vessel cross-sectional area per unit of time. It is a unitless parameter [22,23].

### Statistical Analysis

Data were exported from the Cirrus 5000 device to an Excel file (Excel version 2016; Microsoft Corp., Redmond, WA, USA). In order to ensure the independence of observations, only the left eye of every patient was considered for investigation. Then, data were analyzed with SPSS software (IBM, Chicago, IL, USA, version 24). The Fisher-exact test was used to compare the difference in group proportions relating to sex. The Mann–Whitney U test was used to analyze the differences of continuous parameters between groups. Correlations were calculated by the Spearman correlation test. Data were expressed as mean ± standard deviation. Significance level was *p* < 0.05.

## 3. Results

Fourteen eyes of 14 high-functioning ASD subjects and 14 eyes of 14 age- and sex-matched NT controls were initially examined. One ASD patient did not achieve acceptable signal strength in the scans, so she was discarded. Finally, 13 participants (10 men and 3 women) in the ASD group and 14 (10 men and 4 women) in the NT group were considered (*p* = 1, Fisher-exact test)**.** The mean ages of the ASD and NT groups were 16.615 ± 2.987 and 16.857 ± 4.055 years (*p* = 0.806, Mann–Whitney U test).

**Figure 6:**
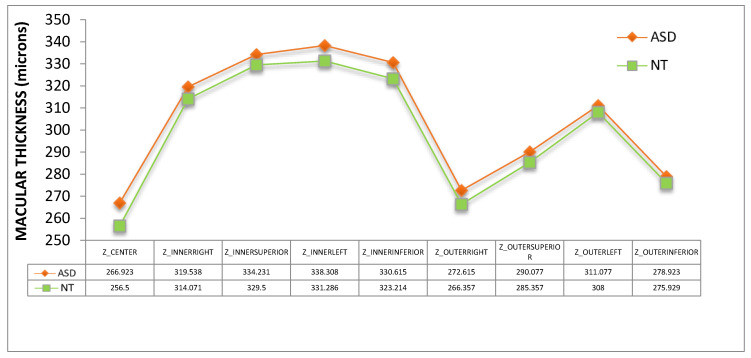
Retinal thickness of the nine subfields of the macular ETDRS grid. ASD = autism spectrum disorder group; NT = neurotypical group. Values represent mean thickness in microns for each group. No significant differences were detected between groups (Mann–Whitney U test).

**Figure 7:**
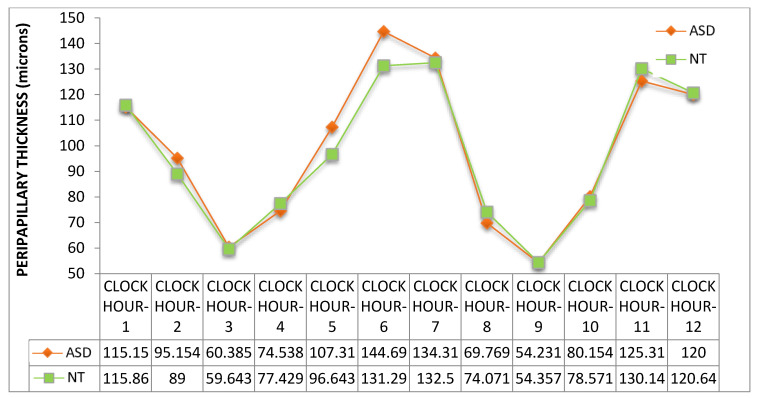
Peripapillary retinal nerve fiber layer thickness of the 12 clock-hour sectors. ASD = autism spectrum disorder group; NT = neurotypical group. Values represent mean thickness in microns for each group. No significant differences were detected between groups (Mann–Whitney U test).

**Figure 8:**
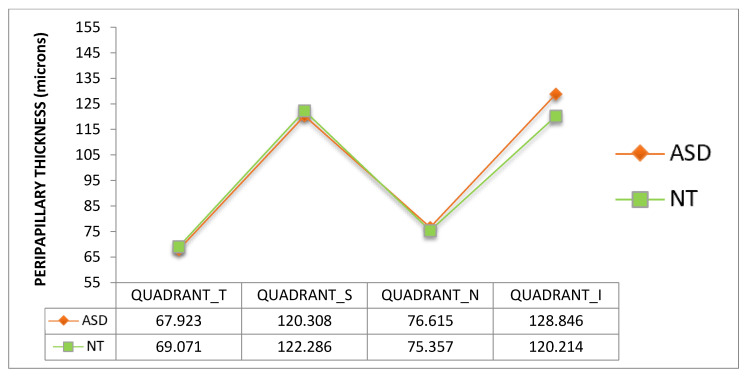
Peripapillary retinal nerve fiber layer thickness of the 4 quadrants. ASD = autism spectrum disorder group; NT = neurotypical group. Values represent mean thickness in microns for each group. No significant differences were detected between groups (Mann–Whitney U test). T = temporal, S = superior, N = nasal, I = inferior.

**Figure 9:**
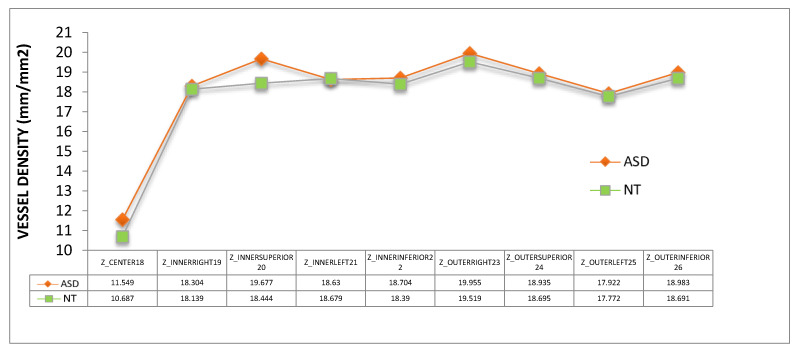
Vessel density of the nine subfields of the macular ETDRS grid. ASD = autism spectrum disorder group; NT = neurotypical group. Mean values were indicated for each group. No significant differences were detected between groups (Mann–Whitney U test).

Thickness of macular (Figure 6 and Figure 7) and peripapillary (Figure 8 and Figure 9) subfields showed no significant differences (*p* > 0.05, Mann–Whitney U test). However, a clear trend to higher thicknesses was found in the ASD group, at all macular subfields and at inferior peripapillary sectors, with such differences as over 10 microns at the macular central subfield (Figure 6), over 13 and 10 microns at clock-hour 6 and 5 sectors, respectively (Figure 7), and over eight microns at inferior peripapillary quadrant (Figure 8).

**Figure 10:**
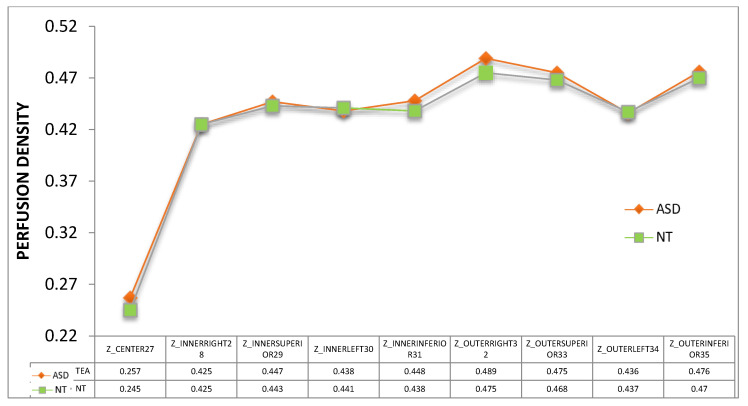
Perfusion density of the nine subfields of the macular ETDRS grid. ASD = autism spectrum disorder group; NT = neurotypical group. Mean values were indicated for each group. No significant differences were detected between groups (Mann–Whitney U test).

Vessel density (Figure 9) and perfusion density (Figure 10) in the macula of the ASD group also showed a tendency to be higher than those of NT group. All these differences were not statistically significant (*p* > 0.05).

FAZ area (ASD = 0.195 ± 0.59 versus NT = 0.194 ± 0.59 mm^2^), FAZ perimetry (ASD = 1.759 ± 0.30 versus NT = 1.652 ± 0.61 mm) and FAZ circularity (ASD = 0.778 ± 0.52 versus NT = 0.727 ± 0.12) differences were not found statistically significant between the ASD and NT groups (*p* > 0.05, Mann–Whitney U test).

**Figure 11:**
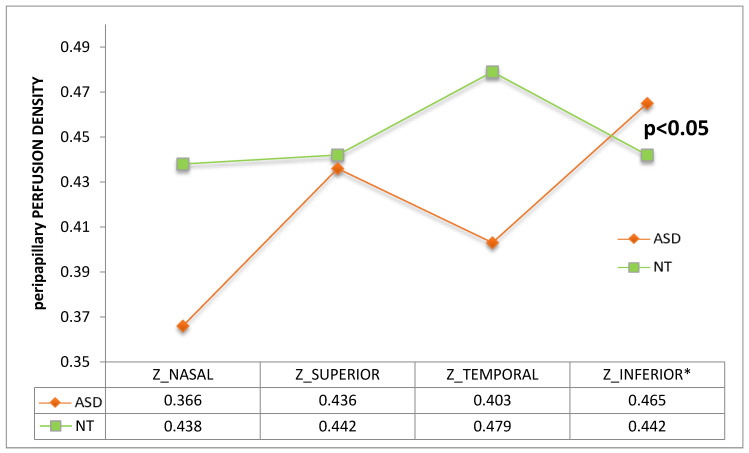
Perfusion density of the 4 peripapillary quadrants. ASD = autism spectrum disorder group; NT = neurotypical group. Mean values were indicated for each group. * Note that the difference between inferior values is statistically significant (*p* = 0.029, Mann–Whitney U test). T = temporal, S = superior, N = nasal, I = inferior.

**Figure 12:**
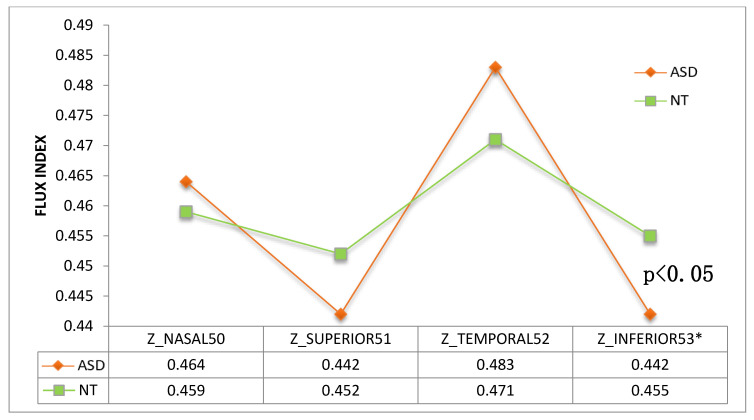
Flux index of the 4 peripapillary quadrants. ASD = autism spectrum disorder group; NT = neurotypical group. Mean values were indicated for each group. * Note that the difference between inferior values is statistically significant (*p* = 0.037, Mann–Whitney U test).

When analyzing ONH perfusion parameters, peripapillary perfusion density was found to be higher (Figure 11) and flux index lower (Figure 12) in the ASD group at the inferior quadrant (*p* = 0.029 and *p* = 0.037 respectively, Mann–Whitney U test).

**Figure 13:**
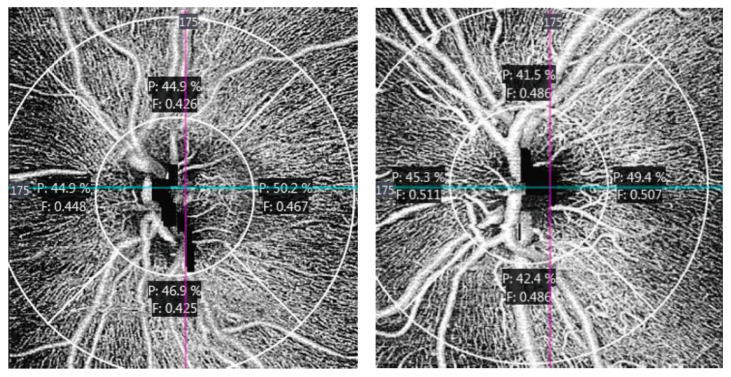
ASD (**left**) and NT (**right**) optic nerve head OCTA of two age and sex-matched participants in this study. Note that perfusion (P) is higher and flux index (F) is lower at the inferior quadrant in ASD participant.

Figure 13 shows an example of ONH OCTA from ASD and NT participants.

**Figure 14:**
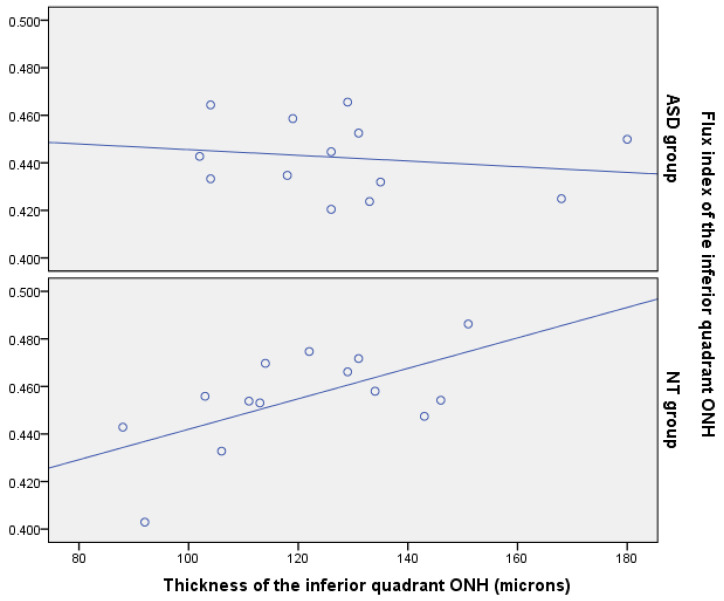
Scatterplots representing the association between peripapillary retinal nerve fiber layer (RNFL) thickness and flux index at the inferior peripapillary quadrant in autism spectrum disorder (ASD) group and neurotypical group (NT). Each point represents the left eye value of each patient. ONH = optic nerve head. Best correlation lines have been added.

Correlations between the perfusion density or flux index and the RNFL thickness at the inferior ONH quadrant were also calculated. Correlations between perfusion density and RNFL thickness were not statistically significant for the ASD or NT group (r = −0.094, *p* = 0.761 and r = 0.103, *p* = 0.725, respectively, Spearman correlation test). However, the correlation of the RNFL thickness and flux index was significant for the NT group (r = 0.591, *p* = 0.029, Spearman correlation test) but not for the ASD group (r = −0.215, *p* = 0.481, Spearman correlation test) (Figure 14).

## 4. Discussion

In this study with OCT and OCTA, we reported a general trend towards a retinal thickening and an increased vascular density in ASD with significant differences in the vessels at the inferior peripapillary ONH quadrant.

Retinal thickness differences in ASD were found previously by our group [17]. In the present study we have found similar results in relation to retinal thickness in a new series of patients and using another OCT device (in our previous study we used a Spectralis OCT device, Heidelberg Engineering, Germany) although the differences have not reached statistical significance. This fact might be due to the small sample size. As examples, we found in our previous study retinal thickenings in the ASD group of 11.6 microns at the center of the macula (*p* < 0.05) or 12.3 microns at the inferior peripapillary quadrant (*p* < 0.05) and in this study the thickenings at these locations were 10.42 (*p* = 0.13) or 8.6 microns (*p* = 0.49), respectively (Figure 6 and Figure 8). These thickenings in ASD could be due to atypical parenchyma overgrowth [24], neuroinflammation [25] or even related to vascular anomalies. For example, disorders of the blood–brain barrier (similar to blood-retina barrier), angiogenesis or endothelial cells have been related to ASD pathophysiology [26,27].

Using OCTA we have detected a general trend towards a higher vessel density and perfusion density in the macula (Figure 9 and Figure 10). However, we found significant results at the inferior peripapillary ONH quadrant. In sum, at this location, perfusion density was higher in ASD (Figure 11), indicating a higher percentage of area occupied by vessels (4.94% more in ASD), and the flux index was lower in ASD (Figure 12), indicating a lower number of red blood cells passing an area per unit of time (2.85% less in ASD). In other words, the vascular density was greater but the flux was less intense in the ASD group at the inferior peripapillary ONH quadrant.

Additionally, we detected that the flux index was positively correlated with thickness at this location in the NT controls but not in ASD individuals. A healthy retina is able to autoregulate the blood flux according to parenchymal metabolic needs [28]. In the NT group the thicker the retinal parenchyma was the higher than the blood flux was, but this phenomenon was not observed in the ASD group (Figure 13). These results suggest that retinal blood flux autoregulation could be altered in ASD.

One may argue that a correction for multiple comparisons should be performed in this study. This is a controversial subject because of the risk that false-negative results could increase [29] so we preferred not to apply such adjustments.

Our work has some limitations. As mentioned above, the small sample size is one of them. However, significant results have been obtained. In addition, OCTA software of the Cirrus device used only provides automated results for superficial capillary plexus but not for deeper vascularization of the retina [30]. Finally, the ASD group was made up of young, high-functioning ASD subjects so the outcomes should not be extrapolated to other groups such as older ASD or low-functioning ASD individuals.

In summary, this pilot study is the first one analyzing retinal parameters in ASD using OCT and OCTA. Retinas of ASD subjects may present some structural and vascular differences when compared with retinas of NT individuals. Further prospective studies are needed to confirm these findings.
